# Structural insights into ChpT, an essential dimeric histidine phosphotransferase regulating the cell cycle in *Caulobacter crescentus*

**DOI:** 10.1107/S1744309112033064

**Published:** 2012-08-29

**Authors:** Antonella Fioravanti, Bernard Clantin, Frédérique Dewitte, Zoé Lens, Alexis Verger, Emanuele G. Biondi, Vincent Villeret

**Affiliations:** a Interdisciplinary Research Institute, USR 3078 CNRS – Université Lille Nord de France, Parc CNRS de la Haute Borne, 50 Avenue de Halley, 59658 Villeneuve d’Ascq, France

**Keywords:** bacterial cell cycle, *Caulobacter crescentus*, histidine kinases, histidine phosphotransferases

## Abstract

Two-component and phosphorelay signal-transduction proteins are crucial for bacterial cell-cycle regulation in *Caulobacter crescentus*. ChpT is an essential histidine phosphotransferase that controls the activity of the master cell-cycle regulator CtrA by phosphorylation. Here, the 2.2 Å resolution crystal structure of ChpT is reported. ChpT is a homodimer and adopts the domain architecture of the intracellular part of class I histidine kinases. Each subunit consists of two distinct domains: an N-terminal helical hairpin domain and a C-terminal α/β domain. The two N-terminal domains are adjacent within the dimer, forming a four-helix bundle. The ChpT C-terminal domain adopts an atypical Bergerat ATP-binding fold.

## Abstract 2

The cell-cycle regulator ChpT of *C. crescentus* is a dimeric histidine phosphotransferase that resembles the typical structure of histidine kinases.

## 1. Introduction

Cell-cycle progression in *Caulobacter crescentus* is coordinated by a complex regulatory network that allows each cell division to produce two distinct cell types: a vegetative and motile daughter cell (named swarmer) and a replicative and sessile mother cell (named stalked). This essential cellular function is mainly controlled by two-component and phosphorelay system proteins (Purcell *et al.*, 2008 ▶). At the core of this circuit resides CtrA, a response regulator that is able to bind DNA and act as a transcription factor, activating and repressing many essential genes involved in crucial cell-cycle steps such as cell division, DNA replication and the development of polar structures (Quon *et al.*, 1996 ▶; Laub *et al.*, 2002 ▶).

As for most response regulators (RRs), activation of CtrA depends on its phosphorylation at a conserved aspartate residue. Phosphoryl­ation of CtrA is carried out by a phosphorelay composed of the membrane hybrid histidine kinase (HK) CckA (Jacobs *et al.*, 1999 ▶) and the histidine phosphotransferase ChpT (Biondi, Reisinger *et al.*, 2006 ▶).

Histidine phosphotransferases (HPTs) are usually monomeric proteins that form a typical structure named a four-helix bundle (Biondi, Skerker *et al.*, 2006 ▶; Xu *et al.*, 2009 ▶). In *Bacillus subtilis*, however, the four-helix bundle structure of Spo0B is achieved by the parallel association of the helical hairpins of two subunits (Varughese *et al.*, 1998 ▶).

ChpT does not belong to any class of known HPTs. Its N-terminal histidine 33 (His33) is the residue that shuttles the phosphate, while the C-terminus weakly resembles an ATP-binding domain (Biondi, Reisinger *et al.*, 2006 ▶). This organization suggests a possible similarity to histidine kinase structures. However, previous biochemical studies have shown that ChpT has no capability to autophosphorylate its His33 using ATP (Biondi, Reisinger *et al.*, 2006 ▶; Chen *et al.*, 2009 ▶), suggesting other roles for the C-terminal domain. Despite the importance of ChpT in cell-cycle regulation in *C. crescentus* and its novelty in the signal transduction field, no structural analysis has been carried out to date.

Here, we report the purification and crystallization of a His_6_-tagged version of ChpT from *C. crescentus*. Its structure was solved, revealing similarities to histidine kinases, with a four-helix bundle flanked by two domains displaying a peculiar ATPase fold.

## 2. Material and methods

### 2.1. Expression and purification of His_6_-ChpT

**Figure 1:**
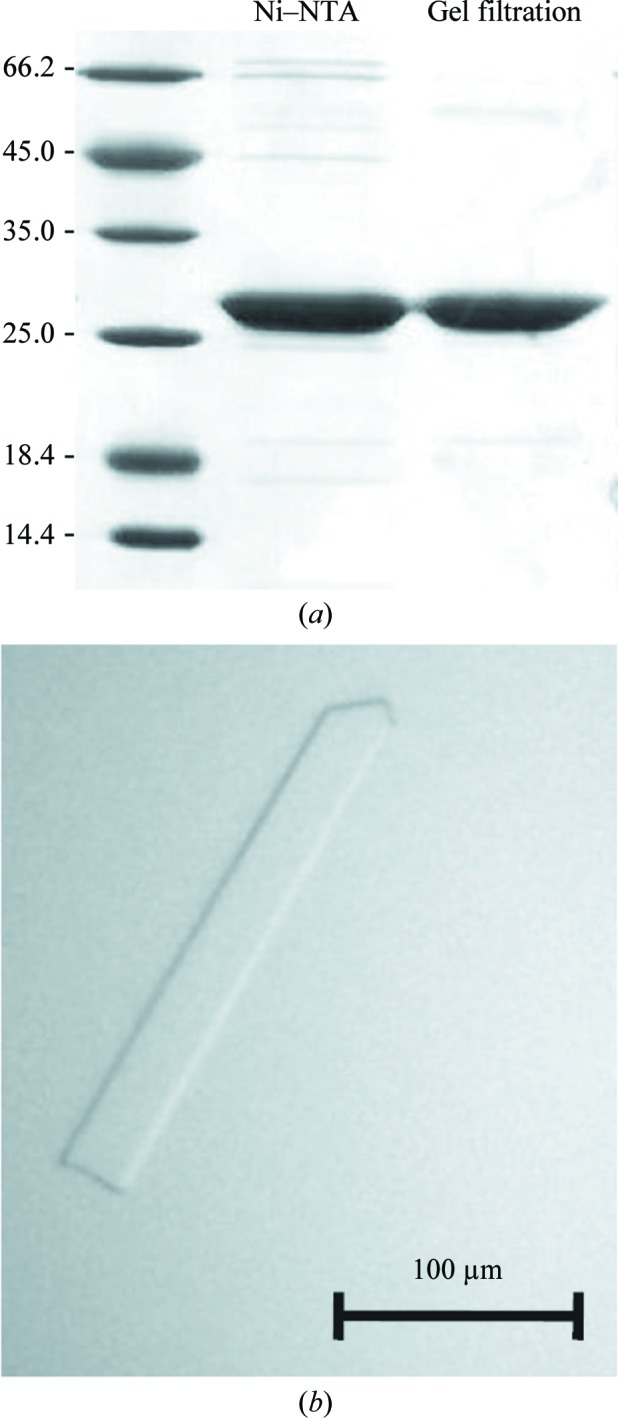
(*a*) SDS–PAGE gel of purified His_6_-ChpT (25.3 kDa) after nickel-affinity purification (Ni–NTA) and gel filtration as described in §[Sec sec2]2. (*b*) His_6_-ChpT crystal.

The full-length DNA fragment of *chpT*, previously annotated CC3470 (Chen *et al.*, 2009 ▶), was amplified using the primers pCC3470-CACC-fw (5′-CACCTTGACCGAGACCGTCACC-3′) and pCC3470-rev (5′-GGTTAAGGAGCGGTTTGCTA-3′) cloned into pENTR/D (Life Technologies, Carlsbad, California, USA) using Gateway technology, sequence-verified and transferred by LR clonase II reaction into pET300/NT-DEST vector (Life Technologies), producing a recombinant IPTG-inducible gene that is able to express ChpT fused to an N-terminal His_6_ tag (MHHHHHHITSL­YKKAG–). Overexpression of His_6_-ChpT (25.29 kDa) was induced in *Escherichia coli* BL21 (DE3) cells at an OD_600 nm_ of 0.6 by the addition of 100 µ*M* isopropyl β-d-1-thiogalactopyranoside (IPTG) and cell growth was continued overnight at 293 K with shaking (215 rev min^−1^). The cells were harvested by centrifugation for 20 min at 500 rev min^−1^ and 277 K, resuspended in lysis buffer [50 m*M* NaH_2_PO_4_, 300 m*M* NaCl, 10 m*M* imidazole pH 8, 1 m*M* DTT, 0.1% Triton X-100, Complete Inhibitor Cocktail (Roche, Basel, Switzerland) and DNase I (Euromedex, Sauffelweyersheim, France)] and lysed using an Emulsiflex homogenizer (Avestin, Ottawa, Ontario, Canada) at 283 K. The supernatant containing His_6_-ChpT was purified using Ni^2+^–nitrilotriacetate (Ni–NTA) affinity resin (Qiagen, Hilden, Germany) equilibrated with lysis buffer and was eluted with NPI-500 pH 8 (50 m*M* NaH_2_PO_4_, 300 m*M* NaCl, 500 m*M* imidazole). A second step of purification was performed by gel filtration using a HiLoad 16/60 Superdex 75 prep-grade column (GE Healthcare, Waukesha, Wisconsin, USA) equilibrated with running buffer [20 m*M* HEPES pH 7.5, 150 m*M* NaCl, 1 m*M* DTT, 10%(*w*/*v*) glycerol]. An SDS–PAGE of His_6_-ChpT purification is shown in Fig. 1 ▶(*a*).

A thermal shift assay (TSA) was performed to optimize the buffer composition as described previously (Niesen *et al.*, 2007 ▶). 24 buffer conditions based on sodium acetate, sodium phosphate, Tris, HEPES, Bicine and potassium phosphate at different concentrations and pH values were assayed (data not shown). His_6_-ChpT was added to a final concentration of 56 µ*M* in a 40 µl total reaction volume containing 5× SYPRO Orange dye and measurements were made using a Mx30005P real-time PCR instrument (Stratagene-Agilent, Santa Clara, California, USA), raising the temperature from 298 to 368 K in 1 K intervals. The optimized buffer composition was 20 m*M* HEPES pH 7.5, 150 m*M* NaCl, 1 m*M* DTT, 10%(*w*/*v*) glycerol.

### 2.2. Crystallization

Initial crystallization trials of His_6_-ChpT (0.5 m*M*) were performed by the sitting-drop vapour-diffusion method using the Cryos Suite kit (Qiagen, Hilden, Germany). The best crystallization hit was obtained in condition No. 88, consisting of 24%(*w*/*v*) polyethylene glycol (PEG) 4000, 0.16 *M* MgCl_2_, 0.08 *M* Tris–HCl pH 8.5, 20%(*w*/*v*) glycerol. These conditions were subsequently refined using the hanging-drop vapour-diffusion method at 293 K in 24-well plates (Hampton Research, Aliso Viejo, California, USA). Drops were prepared by mixing equal volumes (1 µl each) of protein solution and reservoir solution. 0.6 ml precipitant solution consisting of 15%(*w*/*v*) PEG 4000, 0.16 *M* MgCl_2_, 0.08 *M* Tris–HCl pH 8.5, 10%(*w*/*v*) glycerol was pipetted into the reservoir well. The crystal (obtained after 30 d at 293 K) used for data collection is shown in Fig. 1 ▶(*b*).

For crystallization trials with putative substrates, His_6_-ChpT was incubated at 293 K for 30 min in the presence of ATP or ADP (2.5 m*M*) and crystals were obtained under similar conditions.

For experimental phasing, a heavy-atom-derivative crystal was prepared by soaking a crystal for 16 min at 291 K in the crystallization buffer described above with the PEG 4000 concentration increased to 22%(*w*/*v*) in the presence of 100 m*M* Eu-DO3A (europium chelated to 1,4,7,10-tetraazacyclododecan-1,4,7-triacetic acid) obtained from NatX-ray (Girard *et al.*, 2003 ▶). For the native crystal, we used the crystallization buffer with PEG 4000 increased to 20%(*w*/*v*) as a cryoprotectant. The native and heavy-metal-derivative crystals were picked up in a nylon loop and flash-cooled (Niesen *et al.*, 2007 ▶).

### 2.3. Data collection and processing

Data sets were collected from native (His_6_-ChpT-Native) and europium-derivative (His_6_-ChpT-Eu-DO3A) crystals at 100 K on the PROXIMA1 beamline at the SOLEIL synchrotron (Gif-sur-Yvette, France) and BM30 at the European Synchrotron Radiation Facility (Grenoble, France), respectively. The data were processed with *XDS* (Kabsch, 2010 ▶). Data-collection statistics for both data sets are presented in Table 1 ▶.

### 2.4. Structure determination and refinement

The structure was solved by the single isomorphous replacement method with anomalous scattering (SIRAS) using *SHARP*/*autoSHARP* (Vonrhein *et al.*, 2007 ▶). The resolution cutoff used was 3 Å, as suggested by *SHELXC* during the *autoSHARP* procedure. The substructure was subsequently determined by *SHELXD* based on the anomalous signal (Sheldrick, 2008 ▶).

13 Eu-DO3A sites were found in the asymmetric unit, with estimated occupancies of between 20 and 100%. The initial phases were improved using density modification and phase extension by solvent flipping with the program *SOLOMON* (Abrahams & Leslie, 1996 ▶) implemented in *autoSHARP*. Model building was performed using the program *ARP*/*wARP* v.7.1 (Langer *et al.*, 2008 ▶) coupled to the *CCP*4 package (Winn *et al.*, 2011 ▶), specifically using *REFMAC*5 (Murshudov *et al.*, 2011 ▶). The structure was manually completed using the program *Coot* (Emsley *et al.*, 2010 ▶) alternating with cycles of refinement. The final refinement statistics are presented in Table 1 ▶.

## 3. Results and discussion

Data processing revealed that the crystals belonged to space group *C*222_1_, with unit-cell parameters *a* = 103.5, *b* = 210.2, *c* = 93.9 Å for His_6_-ChpT-Native and *a* = 106.4, *b* = 210.0, *c* = 94.1 Å for His_6_-ChpT-Eu-DO3A. Phasing by molecular replacement using known structures of HKs was unsuccessful. Therefore, we solved the His_6_-ChpT structure using a heavy-atom derivative. 13 Eu-DO3A sites were found in the asymmetric unit of the derivative, with occupancies ranging from 20 to 100%.

There are three His_6_-ChpT subunits in the asymmetric unit of the crystal, corresponding to a Matthews coefficient of 3.3 Å^3^ Da^−1^ and an estimated solvent content of 62.8%. Two subunits form a homodimer, while the third subunit exploits a twofold symmetry axis of the lattice to generate a similar homodimer but with exact twofold symmetry. The structure has been deposited in the Protein Data Bank with code 4fpp. The presence of metal ions was suggested in the electron-density map. A fluorescence spectrum measured on the BM30 beamline allowed us to show that the only metal ions present in the cooled sample were nickel ions, which are presumably derived from the purification procedure.

**Figure 2:**
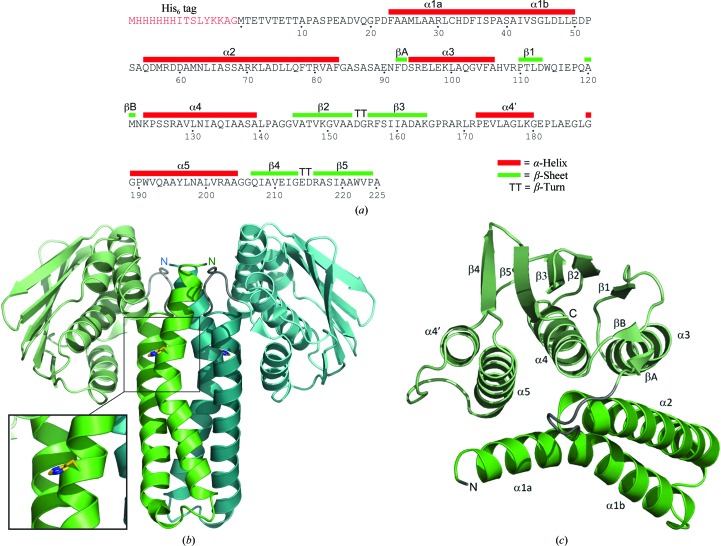
(*a*) Definition of secondary-structure elements in the His_6_-ChpT sequence. (*b*) Ribbon representation of the overall ChpT structure viewed perpendicular to the twofold symmetry axis. The two ChpT subunits are displayed in green and blue, respectively. This view shows the formation of the four-helix bundle of the DHp domain flanked by the two C-terminal domains. The catalytic His33 residues are shown in ball-and-stick representation on opposite faces of the DHp domain. The two N-termini are also indicated. (*c*) Representation of one ChpT subunit, with all secondary-structure elements labelled.

His_6_-ChpT adopts the overall domain architecture of the intra­cellular part of a class I histidine kinase (HK; Fig. 2 ▶). Each subunit consists of two distinct domains, an N-terminal helical hairpin domain and a C-terminal α/β domain, which are connected by a short linker (residues 84–91). The helical hairpin domain is comprised of residues 20–83 and its two antiparallel α-helices are connected by an eight-residue turn (residues 51–58). The N-terminal residues 1–19 are disordered and were not included in the structure.

The dimer interface is exclusively between the two helical hairpin domains and the twofold symmetry axis runs parallel to the helices such that the N-termini are adjacent, forming a four-helix bundle referred to as the dimerization and phosphotransfer (DHp) domain. The DHp domain contains the two H-boxes, with each catalytic His33 located on an opposite face of the four-helix bundle. Helix α1 extends for about 40 Å from the N-terminus to residue 50, displaying a kink induced by Pro38 at the end of the H-box (helix α1, 20–38; helix α2, 39–50). Helix α2 has a similar extension but without any pronounced kink. The four-helix bundle is stabilized mainly by hydrophobic interactions involving Leu27, Leu31, Phe35, Ala39, Ile42, Leu46, Met62, Ala69, Leu72 and Leu76.

**Figure 3:**
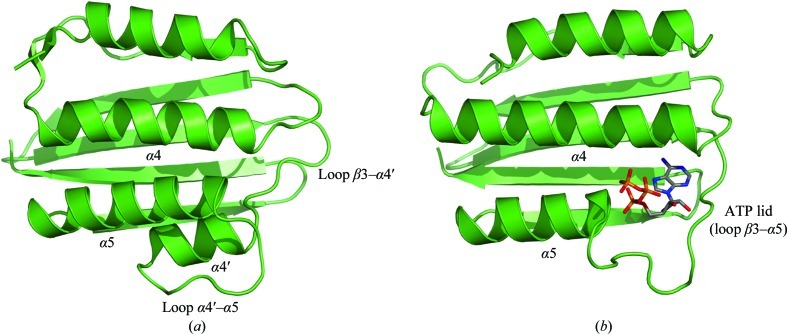
Ribbon representation of the C-terminal domains of (*a*) ChpT and (*b*) DesK (Trajtenberg *et al.*, 2010 ▶). DesK binds ATP in the ATP-binding pocket defined by the ATP lid (a loop between strand β3 and helix α5) and the central helix α4, while the γ-phosphate is exposed and can be attacked by the catalytic histidine of the DHp domain. In ChpT the ATP lid is substituted by two additional turns in the N-terminal part of helix α5. There is an additional short α-helix (α4′) between strand β5 and helix α5, which together with helix α5 closes the ATP-binding pocket.

A structure-similarity search using the *PDBeFold* protein-structure comparison service at the European Bioinformatics Institute (http://www.ebi.ac.uk/msd-srv/ssm; Krissinel & Henrick, 2004 ▶) revealed that the His_6_-ChpT C-terminal (CCT) domain is a close structural homologue of the ATP-binding domain of DesK (r.m.s.d. of 2.15 Å for 109 structurally aligned Cα atoms) (Trajtenberg *et al.*, 2010 ▶), among many other homologous domains belonging to various sensor-type histidine kinases (Fig. 3 ▶). It adopts a Bergerat ATPase fold (Dutta & Inouye, 2000 ▶), which consists of an α/β sandwich with one layer made up of a mixed five-stranded β-sheet and the other layer consisting of three α-helices (α3–α5). In addition, this domain contains a pair of short antiparallel β-strands (βA and βB). It has been shown that ChpT has no capability to autophosphorylate His33 using ATP (Biondi, Reisinger *et al.*, 2006 ▶). The ChpT crystal structure reveals that the CCT domain differs structurally when compared with ATPase domains found in HKs. In HKs with autokinase activity each ATPase domain characteristically hosts one ATP molecule between the ATP lid (a loop between strand β3 and helix α5) and the central helix α4, while the γ-phosphate is exposed and can be attacked by the catalytic histidine of the DHp domain. The bottom of the ATP pocket consists of β-strands 3, 4 and 5. In His_6_-ChpT the ATP lid is substituted by two additional turns in the N-­terminal part of helix α5. These turns occupy the space in which the β- and γ-phosphates are usually located in the CA domain of HKs. Also, there is an additional short α-­helix (α4′) between strand β5 and helix α5 in ChpT, which together with the latter helix closes the ATP-binding pocket found in the CA domain of HKs. We also obtained crystals of His_6_-ChpT in the presence of 2.5 m*M* ATP or ADP in the crystallization buffer. Analyses of these crystals did not reveal any ATP or ADP bound to His_6_-ChpT, which was consistent with the different conformation of the CCT domain (data not shown).

Even if the His_6_-ChpT homodimer shares the overall architecture of class I HK, with its four-helix bundle flanked by two ATP-binding-like domains, significant differences are observed in the relative orientation of these domains. In His_6_-ChpT the two domains adopt a compact conformation, with helices α3, α4 and α5 from the CCT domain lying along helices α1 and α2, leaving the five-stranded β-­sheet of the CCT domain roughly parallel to the helical axis of helix α1. The KinB kinase from *Geobacillus stearothermophilus* (Bick *et al.*, 2009 ▶) is one of the closest structural homologues of ChpT. Although the ChpT and KinB DHP domains superimpose reasonably well (2.1 Å r.m.s.d. on 52 structurally aligned Cα atoms), the disposition of the C-­terminal domains with respect to the DHp domains is very different. Structural alignment of the CCT domain with that of KinB would require an approximate rotation of 55° initiated at the interdomain linker and an outward translation of 20 Å away from the homodimer twofold axis. Structural studies have reported that the CA domains in HKs adopt different positions with respect to the phosphoacceptor His residue according to the step of the phosphotransfer process (Marina *et al.*, 2005 ▶). ChpT is devoid of autokinase activity and thus such specific movements between domains are not expected in the context of an autophosphorylation process. However, it cannot be excluded that domain movement still occurs, for example in the partner-recognition process.

A key characteristic of two-component systems is the high specificity of the HK–RR interaction (Laub & Goulian, 2007 ▶). Because of their structural similarities, we assume that the molecular basis of ChpT–target recognition should be similar to that of HK–RR. Structural insight into HK–RR interactions has recently been revealed by studies of the ThkA–TrrA and HK853–RR468 complexes of *Thermotoga maritima* (Marina *et al.*, 2005 ▶; Casino *et al.*, 2009 ▶, 2010 ▶; Yamada *et al.*, 2009 ▶) and specificity rewiring of TCSs (Skerker *et al.*, 2008 ▶; Capra *et al.*, 2010 ▶; Ashenberg *et al.*, 2011 ▶). These studies showed that the recognition domain of the response regulator (RR) binds to its partner protein *via* interactions with helix α1 of the DHp domain below the phosphodonor His residue and also parts of the ATP lid and the interdomain linker in the HKs. Since the ATP-lid region is severely affected in the ChpT structure by the N-terminal extension of helix α5 and the presence of an additional helix α4′, we anticipate that these differences may have implications for partner recognition of ChpT.

We have demonstrated in this work that His_6_-ChpT, an essential phosphotransferase that controls the phosphorylation of CtrA in *C. crescentus*, adopts the class I histidine kinase fold, with a four-helix bundle flanked by two domains displaying a structurally different ATPase fold. This structure paves the way for future biochemical investigations aiming at deciphering the functional aspects of ChpT regulation and function.

## Supplementary Material

PDB reference: ChpT, 4fpp

## Figures and Tables

**Table 1:** Data-collection and refinement statistics Values in parentheses are for the last shell.

	His_6_-ChpT-Eu-DO3A	His_6_-ChpT-Native
Data collection		
Unit-cell parameters (Å)	*a* = 106.4, *b* = 210.0, *c* = 94.1	*a* = 103.5, *b* = 210.2, *c* = 93.9
Space group	*C*222_1_	*C*222_1_
Beamline	BM30, ESRF	PROXIMA1, SOLEIL
Wavelength (Å)	1.776075	0.980110
Temperature (K)	100	100
Detector	ADSC Quantum 315r	PILATUS 6M
Crystal-to-detector distance (mm)	177	320
Rotation range per image (°)	1	0.2
Exposure time per image (s)	60	0.2
Images collected	180	1200
Resolution (Å)	47–2.5 (2.6–2.5)	49–2.2 (2.3–2.2)
Unique reflections	70189 (7765)	52206 (6430)
Crystal mosaicity (°)	0.146	0.085
Completeness (%)	99.6 (98.9)	100 (100)
Redundancy	3.7 (3.6)	8.7 (9.2)
〈*I*/σ(*I*)〉	9.8 (2.7)	12.5 (2.9)
*R*_merge_ † (%)	10.1 (52.0)	10.7 (81.8)
*R*_meas_ ‡ (%)	11.8 (60.9)	11.4 (86.9)
Overall CC_anom_	0.35	—
CC_anom_ (3.0 Å cutoff)	0.45	—
Refinement data		
*R*_work_ § (%)		22.79
*R*_free_ ¶ (%)		26.38
Mean *B* (Å^2^)		61.9
No. of non-H atoms		
Protein		4549
Ion		1
Water		159
R.m.s. deviations		
Bond lengths (Å)		0.024
Bond angles (°)		2.034
Ramachandran statistics		
Favoured (%)		97.1
Allowed (%)		2.7
Disallowed (%)		0.2

† *R*_merge_ = 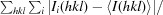 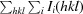, where *I*_*i*_(*hkl*) is the observed intensity and 〈*I*(*hkl*)〉 is the average intensity for multiple measurements.

‡ *R*_meas_ = 100 × 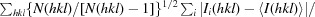 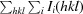, where *N* is the number of times a given reflection has been observed.

§ *R*_work_ = 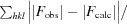 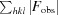, where *F*_obs_ is the observed structure factor and *F*_calc_ is the calculated structure factor.

¶ *R*_free_ is the same as *R*_work_ except calculated using 5% of the data that were not included in any refinement calculations.
